# Hospital Administration

**Published:** 1892-07-09

**Authors:** 


### THE INSTITUTIONAL WORKSHOP.

### HOSPITAL ADMINISTRATION.

### II.—ADMISSION OF PATIENTS.

The admission of patients to the benefits of the charity, their maintenance while there, and their ultimate discharge, being clearly within the objects of the administration, it may be well to define the practice followed by the several types of voluntary hospitals. When first established it was universally the custom, except in cases of accident or extreme urgency, to require a patient to bring with him a recommendation or warrant from a governor or subscriber, who would also be responsible for funeral expenses in the event of death, or removal when called upon. This practice is still followed in most provincial and in all special hospitals, but in the larger general hospitals in London and in several other chief centres of population, patients are received solely on the ground of urgency, curability, or clinical interest, and in keeping with the number of vacant beds at the disposal of the examining officer. The duty of considering whether a patient is eligible or not for admission of course lies with the visiting medical staff, and in their absence with their representatives, holding office as house physicians and house surgeons, and in small hospitals with the resident Medical Officer. In many instances it is customary for the patient to come fortified with a certificate from the local doctor, and, no doubt, this possesses a certain advantage, but the merits of the case can be best judged by the examining officer, who would be guided in his selection by the rules in force in the hospital, together with the accommodation at his command. Where there is a great and incessant demand for the benefits of the charity, as is the case in most metropolitan hospitals of a mixed character, the duties of the medical examiner involve much responsibility, as he has to discriminate between the claims of the poverty stricken, the comparatively well-to-do, and such as are recommended by general practitioners, and by governers of the hospital. This is no easy task at any time, and it becomes doubly difficult in the case of hospitals which receive a large number of accidents. To differentiate between the drunk and dying appears to most people a very simple matter, but from the large contingent of drunken people brought by the police at night to the accident receiving hospitals, it has occasionally happened, notwithstanding much vigilance on the part of the resident officers, that patients have been sent away who were afterwards found to have suffered from head injury of a serious character. It is unreasonable to suppose that the receiving room of a hospital should become a refuge for the dissolute, but on the other hand it is peremptorily necessary in all cases of doubtful diagnosis of the character referred to, that the patient should be retained on the premises, until the cause of the accident is put beyond doubt.

The class of patients admitted to the general as well as to the special hospitals are, as a rule, in a position in life to be able, either of themselves or with the assistance of their friends, to furnish themselves with changes of linen and toilet articles, and in many hospitals, of the poorer class, it is still the custom to require them to provide such articles of food as tea, sugar, and butter, nor is there any special provision made against the introduction of fresh eggs or fruit of a harmless character. There is much to be said in favour of this deviation on the score of self-help, but it unfortunately must place a minority in a somewhat false position, who have not the means of obtaining what is now considered essentially necessary for their comfort, and though in most hospitals the deficiency is made up through the medium of a Samaritan Fund, it is far better that patients entering the establishment should be treated in all respects alike, and furnished with every article of food they require.

From the earliest times it has been deemed necessary in every hospital to possess a diet table specifying the amount of nourishment given to the different classes of patients in accordance with their respective needs. Since each hospital has had to originate its own diet scale, the tables in use vary in construction, but there appears to be a common understanding that this should be limited to three forms, denoting firstly, the full or most generous scale, secondly, the middle or ordinary diet of the establishment, and thirdly, the low diet, which may be limited to milk and beef tea or converted into a vehicle by which extra or fancy articles, such as fish, oysters, or chicken may be prescribed for the patient. The following figures were compiled some years since by the writer from the diet tables of the leading hospitals in the country, and apply to the most generous diet in use in the respective establishments; milk, beer, eggs and gruel are omitted as they are not always sufficiently defined in the diet scales. When reduced to their chemical equivalents of water-free food, the nitrogenous or flesh-forming elements will be found to average between 3 and 4 ozs., and the heat and force producing from 14 to 18 ozs.

| | Bread in oz. | Dressed Meat in oz. | Potatoes in oz. | Pudding in oz. | Mutton broth in oz. | Butter in oz. | Tea & Sugar in oz. |
|---|---|---|---|---|---|---|---|
| St. Bartholomew's | 14 | 8 | 8 | — | — | 1 | 40 |
| Guy's | 14 | 5 | 8 | 4 | 10 | 1 | 20 |
| St. Thomas's | 12 | 4 | 4 | 8 | — | ¾ | 30 |
| The London | 12 | 6 | 8 | 8 | 20 | — | — |
| St. George's | 12 | 6 | 8 | — | — | 1 | 20 |
| The Middlesex | 12 | 8 | 8 | — | 20 | — | — |
| King's College | 12 | 4 | 8 | 8 | — | — | — |
| University College | 12 | 6 | 8 | 4 | 15 | — | — |
| St. Mary's | 15 | 6 | 8 | — | — | ¾ | 40 |
| Westminster | 14 | 6 | 8 | — | — | — | 20 |
| Seamen's | 16 | 8 | 12 | — | 10 | — | 20 |
| Edinburgh Royal | 17 | 6 | 8 | — | 20 | — | — |
| Glasgow Royal | 14 | 4 | 8 | — | 40 | 1 | 32 |
| Manchester Royal | 16 | 6 | 8 | — | — | ¾ | 20 |
| N'wc'stle-on-Tyne | 14 | 6 | 8 | — | 10 | — | 20 |
| Leeds | 16 | 5 | 8 | — | 5 | 1 | 40 |
| Birmingham | 12 | 6 | 8 | — | 20 | — | 20 |
| Dublin | 16 | 8 | 7 | — | 15 | — | 30 |

It is extremely probable that some alterations may have taken place in the daily allowance of food in some of the hospitals since these figures were collected, with the view of varying the monotonous character of the diet, and that the authorities have seen their way to furnish all with a certain amount of butter and fresh vegetables, and fish from time to time. A striking feature of the dietary at present in use in hospitals, is the large amount of milk consumed in comparison with what was formerly the case. In some establishments this amounts to more than a quart a day for each inmate, and in no case does it appear to be under a pint. On the other hand, there has been noticed everywhere a reduction in the consumption of malt liquors, which all must regard as a change for the better, though in most hospitals the wine and spirit bill appears still to keep up its character of extravagance.

Though the dieting of the patients in all establishments is exclusively from the instructions of the medical attendant in the process of distribution, much must be left to the judgment of the nurse superior of the ward, who is best acquainted with the individual tastes and caprices of the patients. It is the custom in most cases that the Sister or head nurse, after the medical visit draws up a diet sheet or score for her patients for the following day, and these coming from all sources to the Secretary's, Matron's, or Housekeeper's departments, according to the usages of the hospital, they are combined and formulated to form the basis of the orders for the following day for the purveyors.

